# Evaluation of a Febrile Neutropenia Protocol Implemented at Triage in an Emergency Department

**DOI:** 10.3390/medicines12030020

**Published:** 2025-08-01

**Authors:** Stefanie Stramel-Stafford, Heather Townsend, Brian Trimmer, James Cohen, Jessica Thompson

**Affiliations:** 1 Department of Pharmacy, Renown Regional Medical Center, Reno, NV 89502, USA; 2 Emergency Department, Renown Regional Medical Center, Reno, NV 89502, USA; 3 Division of Oncology, Renown Regional Medical Center, Reno, NV 89502, USA

**Keywords:** febrile neutropenia, antibiotic therapy, antibiotics, anti-pseudomonal

## Abstract

**Objective:** The impact of a febrile neutropenia (FN) emergency department (ED) triage screening tool and protocol on time to antibiotic administration (TTA) and patient outcomes was evaluated. **Methods:** This was a retrospective, quasi-experimental study of adult FN patients admitted through the ED from April 2014 to April 2017. In March 2016 a triage screening tool and protocol were implemented. In patients who screened positive, nursing initiated a protocol that included laboratory diagnostics and a pharmacy consult for empiric antibiotics prior to evaluation by a provider. Patients were evaluated pre- and post-protocol for TTA, 30-day mortality, ED length of stay (LOS), and hospital LOS. **Results:** A total of 130 patients were included in the study, 77 pre-protocol and 53 post-protocol. Median TTA was longer in the pre-protocol group at 174 min (interquartile range [IQR] 105–224) vs. 109 min (IQR 71–214) post-protocol, *p* = 0.04. Thirty-day mortality was greater at 18.8% pre-protocol vs. 7.5% post-protocol, *p* = 0.12. There was no difference in hospital LOS. Pre-protocol patients compared to post-protocol patients who had a pharmacy consult demonstrated a further reduction in TTA (174 min [IQR 105–224] vs. 87.5 min [IQR 61.5–135], *p* < 0.01) and a reduced mortality (18% vs. 0%, *p* = 0.04). **Conclusions:** To our knowledge, this is the first report of a protocol for febrile neutropenia that allows pharmacists to order antibiotics based on a nurse triage assessment. Evaluation of the protocol demonstrated a significant reduction in TTA and trend toward improved mortality.

## 1. Introduction

### 1.1. Background

Febrile neutropenia (FN) is a life-threatening complication of patients with cancer [1,2]. In the U.S., in 2012 there were 91,560 adult cancer-related neutropenia hospitalizations, with a total cost of USD 2.3 billion and an average of 9.6 hospital days [1]. Since then, for many reasons (increased cancer diagnosis, etc.), these numbers have likely only increased. Additionally, the risk of infection increases with the degree and duration of neutropenia [2,3,4]. Patients with FN have a 30% risk of developing end-organ dysfunction and an 11% risk of mortality [2,3,4]. Furthermore, FN patients with sepsis have a 50% mortality risk [2,3,4]. Clinical guidelines developed by the American Society of Clinical Oncology (ASCO) and the Infectious Diseases Society of America (IDSA) recommend that the first empiric antibiotic dose be administered within one hour of triage in an effort to improve outcomes [2,3]. However, with average emergency department (ED) wait times to see a provider exceeding 15 min, a 60 min time to antibiotic administration (TTA) is very difficult to achieve [5].

### 1.2. Importance

At the time of this study, during our search, only five articles published since 2009 were identified when searching OVID and PubMed for the specific words “febrile neutropenia time to administration”, and all except one were pediatric studies that investigated TTA for patients with FN [6,7,8,9,10,11,12]. Only one adult publication provided a suggested process towards achieving guideline-recommended goals [9]. Therefore, improving time to administration for patients with febrile neutropenia is likely an opportunity for many emergency departments.

### 1.3. Goals of This Investigation

This study was designed to evaluate whether a nursing and pharmacy ED triage screening tool and protocol improves TTA and patient outcomes.

## 2. Methods

### 2.1. Study Design and Setting

This was a retrospective, quasi-experimental study of FN patients who presented to the ED at an 808-bed not-for-profit, community-teaching level II trauma hospital from April 2014 to April 2017. Renown Health receives over 100,000 annual emergency department visits, with over 31,000 annual admissions, and Renown’s Institute for Cancer offers services in surgical, medical, and radiation oncology.

### 2.2. Selection of Participants

**Figure 1:**
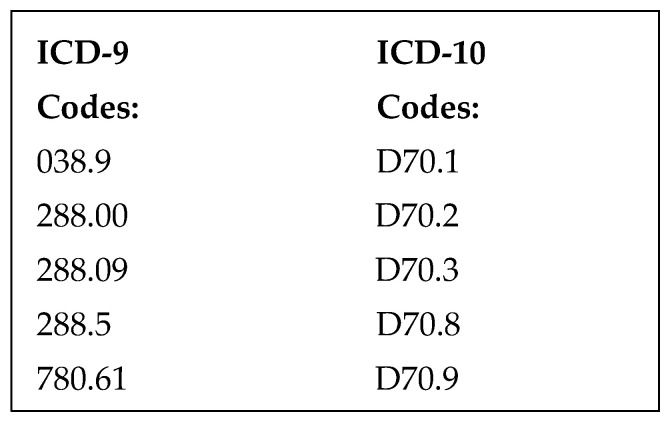
Qualifying ICD codes.

Patients were included in the study if they had an ICD-9 or ICD-10 code for neutropenia, cancer, or chemotherapy (Figure 1) and were admitted to the hospital through the ED with fever and neutropenia. Fever was defined as either ≥100.4 °F (38 °C) or <96.5 °F (35.8 °C) within 24 h of presentation or fever prior to arrival with the use of acetaminophen or NSAIDs. Neutropenia was defined as an absolute neutrophil count (ANC) < 1000 per microliter within 24 h of admission. Patients were excluded if they were <18 years of age, pregnant, or prisoners.

### 2.3. Interventions

**Figure 2:**
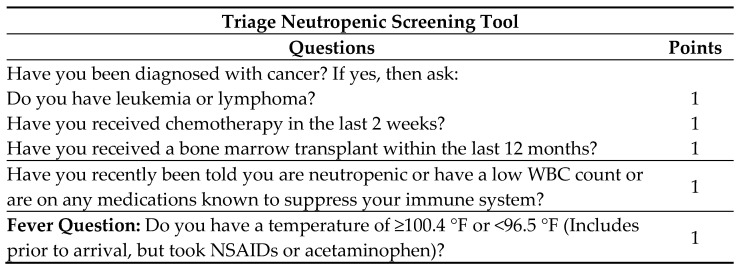
Triage neutropenic screening tool.

**Figure 3:**
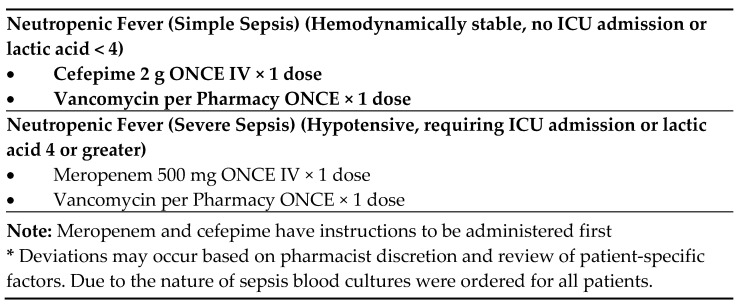
Recommended antibiotics *.

During patient triage in the ED, the neutropenic triage screening tool is completed by nursing (Figure 2). If positive on screening, the protocol order set is initiated. A positive triage screen is a score of 2 or more, with 1 point coming from the question pertaining to fever. The protocol includes orders for cultures from potential infectious sites, diagnostic laboratory tests, and a “nursing communication alert” for nursing to notify the physician and pharmacy of the patient, and for a pharmacy consult to initiate broad-spectrum antibiotics. If no antibiotic orders from the physician are received within 15 min from receipt of the consult, the pharmacist will initiate recommended antibiotics (Figure 3) independently of the physician per protocol. Deviations from recommendations may occur based on pharmacist discretion and review of patient-specific factors. This process occurs roughly 30 min after presentation to the ED. All of this is an effort to reach a TTA of under 60 min.

### 2.4. Data Collection

Data was collected from the electronic medical record. State death certificate data was collected in conjunction with Desert Research Institute, as contracted with Renown Health. Patient baseline characteristics gathered from the EMR included age, weight, height, gender, ethnicity, allergy information, admission diagnosis, ICD-10 and ICD-9 diagnosis, first temperature recorded, maximum temperature recorded during hospital stay, minimum absolute neutrophil count (ANC) recorded, maximum ANC recorded, febrile neutropenia screening score and flowsheet information, source of infection, time to first antibiotic, first antibiotic administered, microbiology results, ED length of stay, ED door-to-doctor time, hospital length of stay, and 30-day mortality.

### 2.5. Outcomes

The primary outcome was time to first antibiotic. This was defined as the time from arrival at the ED until the first antibiotic was administered. Secondary outcomes included: 30-day mortality, ED length of stay, and hospital length of stay.

### 2.6. Statistical Analysis

Statistical analyses were performed with the SAS statistical program. Chi-squared tests and Fisher’s Exact Tests were used for categorical data and Wilcoxon Rank-Sum Tests for continuous variables. A *p* value of <0.05 was considered statistically significant. Power analysis estimated that 139 total patients were required to demonstrate a 30% reduction in time to administration of antibiotics with a 95% confidence interval.

This study was approved by the institutional review board: 1295724-1.

## 3. Results

### 3.1. Characteristics of Study Subjects

**Figure 4:**
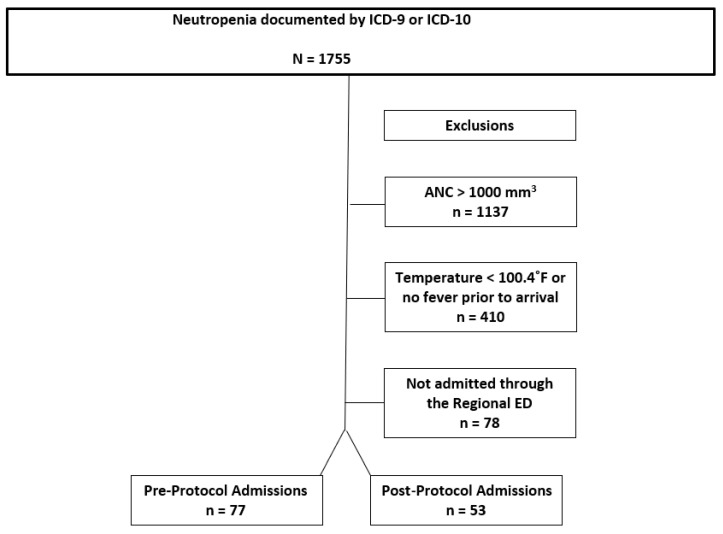
Study flow diagram.

We identified 1755 ED visits by qualifying ICD-9 or ICD-10 codes (Figure 1) from April 2014 to April 2017. There were 1137 patients excluded who were not neutropenic by a priori definition, 410 were excluded because they did not meet fever criteria, and 78 were excluded because they were not admitted through the emergency department (Figure 4). Therefore 130 patients were included in the analysis: 77 as pre-protocol admissions and 53 as post-protocol admissions. The characteristics for all episodes of FN are shown in Table 1. The mean age of our study population was 59 (SD 16) in the pre-protocol and 55 (SD 20) in the post-protocol group. Both groups were predominantly composed of Caucasian males. One notable observed characteristic was that there was a higher percentage (49%) of post-protocol patients with profound neutropenia. Also, the neutropenic triage screen was successfully completed by nursing in 81% of triaged patients. Roughly 30% of the culture results were positive for both groups, and the predominant positive source was blood for both groups. Of note, patients could have more than one organism isolated from more than one source. It was observed that *Pseudomonas aeruginosa* was only identified in four results, MRSA was only identified in one result, and only two results of *Enterococcus* spp. were seen over the 3-year study period.

### 3.2. Main Results

**Figure 5:**
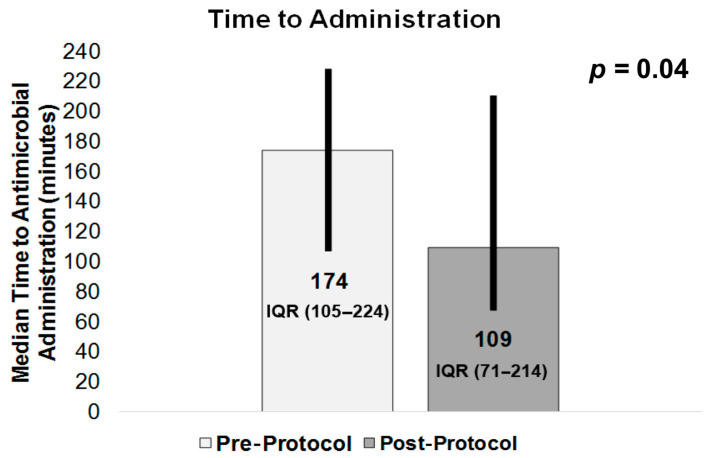
Time to Administration.

TTA, our primary outcome (shown in Figure 5), was 174 min (IQR 105–224) in the pre-protocol group vs. 109 min (IQR 71–214) in the post-protocol group (*p* = 0.04), with TTA assumed a priori to be reduced post-protocol. Among our secondary outcomes, the results for 30-day mortality (Table 2) were 14 deaths (18%) in the pre-protocol group vs. 4 deaths (8%) in the post-protocol group (*p* = 0.12). Median (IQR) hospital length, in days, was 5 (3–8) for the pre-protocol group vs. 4 (3–7) for the post-protocol group (*p* = 0.003). ED median (IQR) length of stay was 301 min (231–339) for the pre-protocol group and 365 min (265–445) for the post-protocol group (*p* = 0.003).

**Figure 6:**
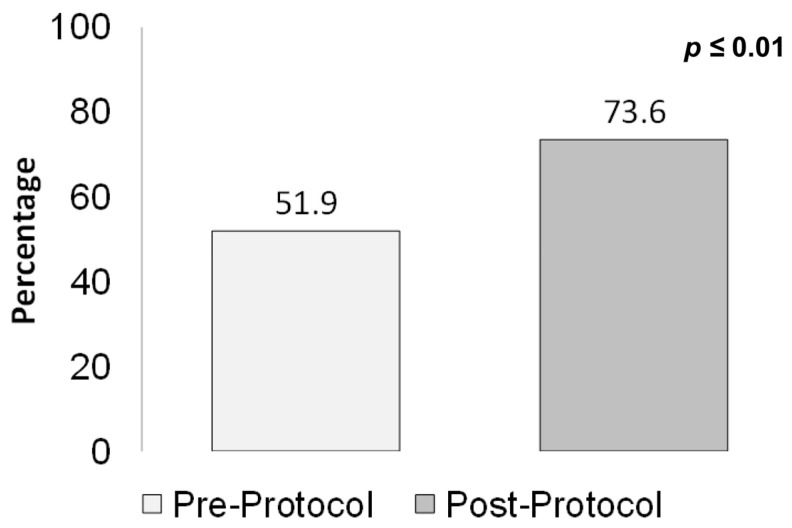
Admissions administered an anti-pseudomonal as a first dose. Other antimicrobials administered were acyclovir, ceftazidime, ciprofloxacin, clindamycin, ertapenem, fluconazole, levofloxacin, metronidazole, and oseltamivir.

The proportion of patients who were administered a first dose of anti-pseudomonal beta-lactams (Figure 6), in accordance with guideline recommendations, was 52% pre-protocol vs. 74% post-protocol (*p* ≤ 0.01).

The data was further analyzed with a subgroup analysis of those patients who screened positive on the triage screen. A positive triage screen was equal to a score of ≥two on the neutropenic triage screening tool. Time to administration in this subgroup analysis, shown in Table 3, was a TTA of 87.5 min in the positive triage screen group vs. 174 min in the pre-protocol group (*p* < 0.01). Additionally, 30-day mortality in this group showed no mortalities in those who screened positive on the triage screen vs. 14 mortalities in the pre-protocol group (*p* = 0.04).

## 4. Limitations

This study had several limitations. Because this study was a single-center retrospective chart review, there was a potential for bias, particularly in the way patients were identified. ICD-9 or ICD-10 code documentation is included in the diagnostic process, and charting may have deviated in some instances, which could have led to fewer patients being identified. Also, the performance of the triage screen relied on patients answering the questions, and the results may have deviated based on education level, ability to communicate, or interpretation/medical understanding of the questions, predominantly by the patient self-reporting, family or friend reporting, or emergency medical services report. The number of false-positive triage screens that may have occurred was also not assessed. This may have led to the inclusion of patients who were not truly febrile, and if they screened positive, then they should not have been included, and their inclusion may have resulted in an overestimation of the consult effect. Further, for this study, the diagnostic performance of the screening tool was not assessed. Thus, a lack of sensitivity could lead to a decrease in the administration of needed prescriptions, while there is a possibility of high rates of false-positive results, which could lead to unnecessary prescriptions and potential secondary effects, toxicities, side effects like *Clostridioides difficile* colitis, antimicrobial resistance development, and/or increased healthcare costs. Further studies that explicitly assess the diagnostic performance of such triage screening tools are needed.

## 5. Discussion

This retrospective, quasi-experimental study on febrile neutropenic patients presenting to the ED allowed for the identification of an effective pharmacy- and nursing-driven protocol utilized at triage to decrease time to administration of antibiotics. In the ED, some of the most difficult tasks in the treatment of these patients are the identification of these patients and the administration of antibiotics within the guideline-recommended time. This study showed a 39% decrease in TTA overall and a 49% reduction in TTA in those who had a positive screen and pharmacy consult. Although not statistically significant, mortality went from 18% pre-protocol to 8% post-protocol. No mortalities were seen in the subgroup that screened positive and received a pharmacy consult, with 18% in the pre-protocol group vs. 0% in those who received a pharmacy consult (*p* = 0.04). Other secondary outcomes were not as impactful, with an increased ED LOS and no difference in hospital LOS. However, the increased ED LOS was thought to be likely due to ED boarding patients during the post-protocol phase.

Documentation, which plays a key role in our protocol, revealed that the neutropenic triage screen was documented in 81% of post-protocol admissions. A review of the 19% of admissions where it was not documented revealed that most appeared to be in the 6-week period just after implementation of the protocol. Furthermore, a review of the negative triage screens (those that did not score ≥2 on the screen, with 1 point being from the fever question) revealed that only 65% were documented accurately. In several instances, this seemed to have occurred due to the completion of the screen before receiving patient information from emergency medical services (as identified as a possible limitation above). This contributed to a decrease in the number of patients receiving pharmacy consults and could have impacted their TTA.

One notable finding in our analysis was that positive culture results revealed that only 2.3% of all patients would have benefited from the administration of vancomycin, with only three patients growing MRSA or *Enterococcus* spp., and empiric administration of vancomycin is not recommended in all patients per IDSA and ASCO guidelines [2,3].

Future directions are aimed at improving the utilization and accuracy of the neutropenic triage screening tool. This includes collaborating with emergency services and nursing education. Also, recently, anti-pseudomonal antibiotics were added to automated dispensing machines in the ED, which will allow for the assessment of the impact on TTA, with a possible further reduction in TTA. Furthermore, criteria are being developed for vancomycin administration because organisms warranting this addition to the protocol were rarely identified. Lastly, updates to the protocol may allow pharmacists to immediately order antibiotics after receipt of the pharmacy consult instead of waiting the previously required 15 min.

In summary, future methods aimed at reducing TTA, inappropriate empiric antibiotics, any delays in provider evaluation, and possible workflow issues, as well as decreasing adverse events in patients with febrile neutropenia, are currently being investigated. No specific nationally utilized protocol has been identified for these patients, and our pre-protocol TTA of 174 min was reduced to 109 min after the implementation of the protocol. A further reduction to 87.5 min was seen with those who screened positive on the triage screen and received a pharmacy consult. Thus, a pharmacist- and nursing-driven protocol significantly improves TTA and will be continued in the ED with the aim of improving patient outcomes for patients with febrile neutropenia.

## Figures and Tables

**Table 1:** Baseline characteristics.

	Pre-Protocol	Post-Protocol
	n = 77	n = 53
**Male, n (%)**	46 (60)	29 (55)
**Mean age, years (SD)**	59 (16)	55 (20)
**Ethnicity, n (%)**	63 (82)	46 (87)
White, n (%)		
**Temperature, °F (SD)**	100.4 (2.0)	100.2 (2.0)
**ANC**		
500 to <1000/µL, n (%)	17 (22)	9 (17)
100 to 499/µL, n (%)	33 (43)	18 (34)
<100/µL, n (%)	27 (35)	26 (49)
**RN triage screening performed, n (%)**	NA	43 (81)
**Positive culture results, n (%)**	22 (29)	15 (28)
**Source of positive culture** *		
Blood, n	11	8
Urine, n	6	5
Respiratory, n	3	5
Other, n	3	1
**Organisms resulted** *		
*Pseudomonas aeruginosa*, n	1	3
ESBL *Escherichia coli*, n	1	1
*Staphylococcus aureus*, n	3	1
MRSA, n	1	0
*Enterococcus* spp., n	1	1
Other, n	18	16

* Patients could have more than one organism isolated from more than one source.

**Table 2:** Secondary outcomes.

Outcome	Pre-Protocol n = 77	Post-Protocol n = 53	*p* Value
**30-day mortality, n (%)**	14 (18)	4 (8)	0.12
**ED LOS, min. median (IQR)**	301 (231–339)	365 (265–445)	<0.01
**Hospital LOS, days median (IQR)**	5 (3–8)	4 (3–7)	0.28

**Table 3:** Positive screen subgroup analysis.

Outcome	Pre-Protocol n = 77	Positive Triage Screen * n = 20	*p* Value
**TTA, min median (IQR)**	174 (105–224)	87.5 (61.5–135)	<0.01
**30-day mortality, n (%)**	14 (18)	0 (0)	0.04

* Patients in the post-protocol group who were positive on the triage screen and received a pharmacy consult.

## Data Availability

The datasets presented in this article are not readily available because of HIPAA. Requests to access the datasets should be directed to Stefanie Stafford-Stramel.
